# Redetermination of dipotassium trichlorido­stannate(II) chloride monohydrate

**DOI:** 10.1107/S1600536813001372

**Published:** 2013-01-19

**Authors:** Fei Ye, Hans Reuter

**Affiliations:** a Institut für Chemie neuer Materialien, Anorganische Chemie II, Universität Osnabrück, Barbarastrasse 7, 49069 Osnabrück, Germany

## Abstract

The title compound, K_2_[SnCl_3_]Cl·H_2_O, is the prototype of some isostructural compounds of composition *M*_2_[Sn*X*_3_]*X*·H_2_O (*M* = large monovalent cation; *X* = halogen). In comparison with a previous study based on photographic data [Kamenar & Grdenić (1962 ▶). *J. Inorg. Nucl. Chem.* **24**, 1039–1045], its crystal structure has now been redetermined using CCD-based data in order to gain more accurate values for bond lengths and angles within the [SnCl_3_]^−^ anion and to locate the H atoms. The [SnCl_3_]^−^ anion has a trigonal–pyramidal shape and exhibits crystallographic mirror symmetry. With the exception of the K^+^ ion which is located on a general position, all other atoms are situated on crystallographic mirror planes. The coordination polyhedron of the cation may be described by means of nine atoms in the form of a monocapped square anti­prism with seven typical K—Cl/O distances and two additional atoms at considerably longer distances. The positions of the H atoms of the water mol­ecule (also lying on a crystallographic mirror plane) could be determined and confirm the existence of a bifurcated O—H⋯Cl hydrogen bond to neighbouring Cl atoms.

## Related literature

For a previous crystallographic investigation of the title compound, see: Kamenar & Grdenić (1962 ▶). For IR-spectra of the title compound, see: Falk *et al.* (1974 ▶).

## Experimental

### 

#### Crystal data

K_2_[SnCl_3_]Cl·H_2_O
*M* *_r_* = 356.71
Orthorhombic, 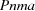

*a* = 11.9233 (9) Å
*b* = 9.0768 (7) Å
*c* = 8.1737 (6) Å
*V* = 884.60 (12) Å^3^
*Z* = 4
Mo *K*α radiation
μ = 4.95 mm^−1^
*T* = 100 K
0.24 × 0.06 × 0.04 mm

#### Data collection

Bruker APEXII CCD diffractometer
Absorption correction: multi-scan (*SADABS*; Bruker, 2009 ▶) *T*_min_ = 0.388, *T*_max_ = 0.823
27095 measured reflections
1367 independent reflections
1265 reflections with *I* > 2σ(*I*)
*R*_int_ = 0.052

#### Refinement

*R*[*F* ^2^ > 2σ(*F* ^2^)] = 0.013
*wR*(*F* ^2^) = 0.028
*S* = 1.12
1367 reflections
45 parameters
H-atom parameters constrained
Δρ_max_ = 0.34 e Å^−3^
Δρ_min_ = −0.48 e Å^−3^

### 

Data collection: *APEX2* (Bruker, 2009 ▶); cell refinement: *SAINT* (Bruker, 2009 ▶); data reduction: *SAINT*; program(s) used to solve structure: *SHELXS97* (Sheldrick, 2008 ▶); program(s) used to refine structure: *SHELXL97* (Sheldrick, 2008 ▶); molecular graphics: *DIAMOND* (Brandenburg, 2006 ▶) and *Mercury* (Macrae *et al.*, 2008 ▶); software used to prepare material for publication: *SHELXTL* (Sheldrick, 2008 ▶).

## Supplementary Material

Crystal structure: contains datablock(s) I, global. DOI: 10.1107/S1600536813001372/wm2717sup1.cif

Structure factors: contains datablock(s) I. DOI: 10.1107/S1600536813001372/wm2717Isup2.hkl

Additional supplementary materials:  crystallographic information; 3D view; checkCIF report

## Figures and Tables

**Table:** 

Sn1—Cl2	2.5745 (3)
Sn1—Cl2^i^	2.5745 (3)
Sn1—Cl1	2.6034 (5)

**Table:** 

Cl2—Sn1—Cl2^i^	87.945 (16)
Cl2—Sn1—Cl1	87.376 (11)
Cl2^i^—Sn1—Cl1	87.376 (11)

Symmetry code: (i) 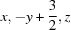.

**Table 2:** Hydrogen-bond geometry (Å, °)

*D*—H⋯*A*	*D*—H	H⋯*A*	*D*⋯*A*	*D*—H⋯*A*
O1—H1⋯Cl3^ii^	0.96	2.34	3.2920 (16)	174
O1—H2⋯Cl2^iii^	0.96	2.61	3.3687 (12)	137
O1—H2⋯Cl2^iv^	0.96	2.61	3.3687 (12)	137

Symmetry codes: (ii) 
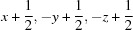
; (iii) 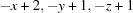; (iv) 
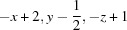
.

**Fig. 1.**
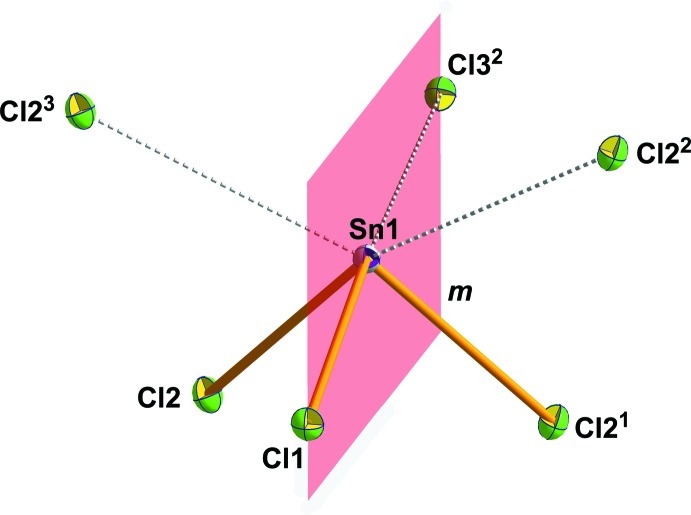
Ball-and-stick model of the [SnCl3]- anion and its surrounding with the atomic numbering scheme and the crystallographic mirror plane indicated (transparent, red); all atoms are represented as displacement ellipsoids at the 70% probability level; covalent bonds are indicated as thick sticks (orange), electrostatic interactions as thin, broken ones (grey). [Symmetry codes: (1) x, 3/2 - y, z; (2) 2 - x, 1/2 + y, -z; (3) 2 - x, 1 - y, -z.]

**Fig. 2.**
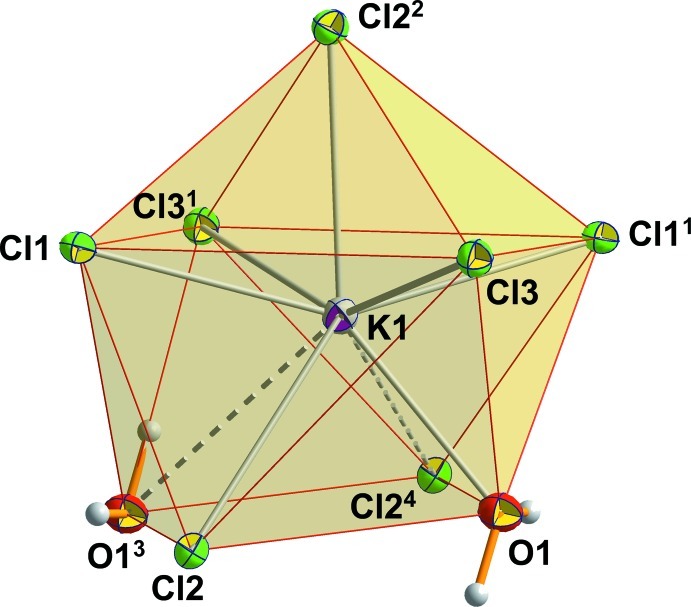
Ball-and-stick model of the coordination sphere of the potassium ion; with exception of the hydrogen atoms, which are shown as spheres of arbitrary radius, all other atoms are represented as displacement ellipsoids at the 70% probability level; covalent bonds of H2O are indicated as thick sticks (orange), strong/weak electrostatic interactions (yellow) as thick unbroken/broken ones. [Symmetry codes: (1) 3/2 - x, 1 - y, 1/2 + z; (2) -1/2 + x, y, 1/2 - z; (3) 2 - x, 1/2 + y, 1 - z; (4) 2 - x, 1 - y, 1 - z.]

**Fig. 3.**
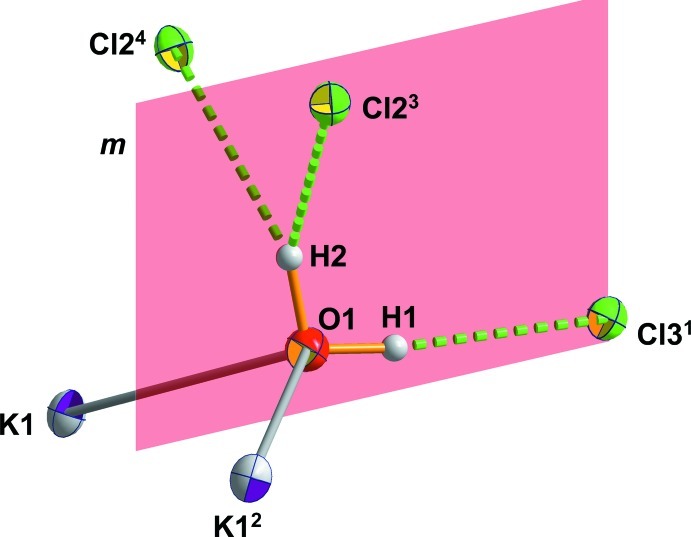
Ball-and-stick model of the water molecule and its hydrogen bonds (green) to chlorine atoms; with exception of the hydrogen atoms, which are shown as spheres of arbitrary radius, all other atoms are represented as displacement ellipsoids at the 70% probability level. [Symmetry codes: (1) 1/2 + x, 1/2 - y, 1/2 - z; (2) x, 1/2 - y, z; (3) 2 - x, -1/2 + y, 1 - z; (4) 2 - x, 1 - y, 1 - z.]

**Fig. 4.**
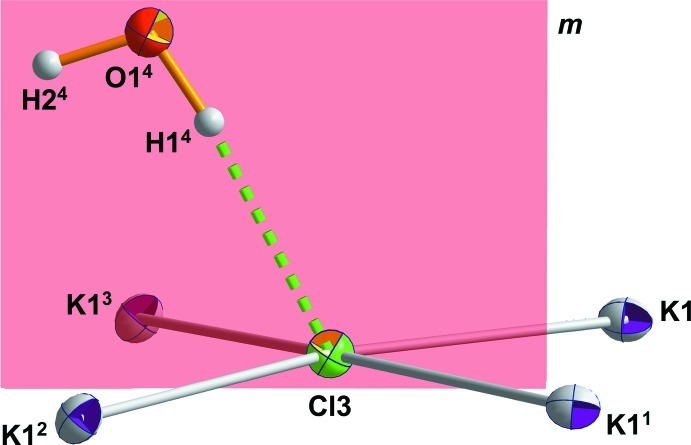
Ball-and-stick model of the coordination mode of the isolated chlorine atom Cl3; with exception of the hydrogen atoms, which are shown as spheres of arbitrary radius, all other atoms are represented as displacement ellipsoids at the 70% probability level. [Symmetry codes: (1) x, 1/2 - y, z; (2) 3/2 - x, -1/2 + y, -1/2 + z; (3) 3/2 - x, 1 - y, -1/2 + z; (4) -1/2 + x, 1/2 - y, 1/2 - z.]

**Fig. 5.**
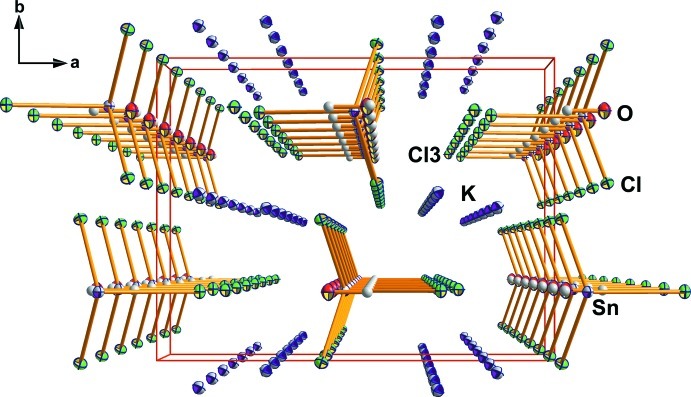
Packing diagram only representing the covalent bonds (yellow) of the trichloridostannate(II) ions and the water molecules; with exception of the hydrogen atoms, which are shown as spheres of arbitrary radius, all other atoms are represented as displacement ellipsoids at the 70% probability level.

## supplementary crystallographic information

### Comment

The title compound came into the focus of our interest when we became aware that it represents the prototype of a series of isostructural compounds of composition *M*_2_[Sn*X*_3_]*X*.H_2_O with *M* = K, Rb, NH_4_; *X* = Cl, Br, containing a trigonal-pyramidal [SnCl_3_]^-^ ion with crystallographic mirror symmetry that is only slightly distorted by weak secondary interactions of the tin(II) atom by more remote neighbouring chlorine atoms. Unfortunately, the precision of bonds lengths and angles available from the literature suffer from the fact that the crystal structure has been determined from two sets of Weissenberg photographs covering only 178 reflections with *hk*0 and 0*kl* (Kamenar & Grdenić, 1962). To get more precise and accurate information, we decided to reinvestigate the crystal structure at a temperature of 100 K to 2θ = 60°. Moreover, we intended to determine the position of the hydrogen atoms in order to confirm an older assumption on a bifurcated hydrogen bond derived from IR data (Falk *et al.*, 1974).

The asymmetric unit of K_2_[SnCl_3_]Cl.H_2_O consists of half a formula unit, with one potassium ion in a general position, half a [SnCl_3_]^-^ ion (with one chlorine atom in a general position and the tin atom as well as a second chlorine atom on the mirror plane), as well as a water molecule and third chlorine atom also located on a mirror plane.

As a result of its *m* symmetry, two of the three bond lengths and bond angles within the [SnCl_3_]^-^ anion are equal (Tab. 1). The two different Sn—Cl bonds are very similar and differ by only 0.028 Å [mean value: 2.584 (17) Å]. All bond angles are significantly smaller than 90° and very similar, too, the difference being 0.57° [mean value: 87.6 (3)°]. All in all, deviations from higher point group symmetry 3*m* are very small. The coordination sphere of the tin atom is augmented by three additional chlorine atoms at more remote distances of 3.2854 (4) Å (Cl2) and 3.1311 (5) Å (Cl3, 2*x*). The interaction must be contributed mainly to electrovalent (ionic) forces although it is worthwhile to see that the shorter distance is opposite to the longest (Cl1) of the two covalent Sn—Cl bonds. In summary, this sixfold coordination gives rise to a strongly distorted octahedral arrangement (Fig. 1).

The potassium ion is surrounded in form of a distorted pentagonal biypramid from 6 chlorine atoms [2 *x* Cl1, 2 *x* Cl2, 2 *x* Cl3] with K—Cl distances in a very narrow range of 0.09 Å [3.1364 (4) - 3.2475 (4) Å, mean value: 3.21 (4) Å] and the oxygen atom of a water molecule in a distance of 2.7960 (10) Å. On the background that Cl1 and Cl2 are covalently bonded to tin it is interesting to see that the two shortest K—Cl distances [3.1364 (4) and 3.1948 (5) Å] are those to the isolated chlorine atom Cl3. The potassium coordination sphere is completed by an additional chlorine atom at a considerable longer distance of 3.7935 (5) Å (Cl3), and an additional oxygen atom of a second water molecule at a distance of 3.908 (1) Å. Taking these additional atoms into account, the coordination polyhedron of the potassium ion is best described as a distorted monocapped square antiprism (Fig. 2).

Since we could determine the position of both hydrogen atoms of the water molecule we were also able to confirm a former assumption on the existence of a bifurcated hydrogen bond of one hydrogen atom to two neighbouring chlorine atoms (Fig. 3). All three atoms of the the water molecule lie on a crystallographic mirror plane. Thus, the oxygen atom is coordinated to two potassium ions (µ_2_-O) outside the mirror plane with two equal distances of 2.7960 (10) Å, the angle between the potassium ions being 97.99 (5)°. One hydrogen atom (H1) forms a normal hydrogen bond to one chlorine atom (Cl3), also situated on the mirror plane, whereas the other one (H2) forms a bifurcated hydrogen bridge to two chlorine atoms (Cl2) outside the mirror plane, the latter being significantly weaker (longer) than the first one (Tab. 2), with both chlorine atoms enclosing an angle of 86.65 (1)° at this hydrogen atom.

The coordination sphere of the chlorine atom not covalently bonded to tin (Cl3), consists (Fig. 4) of four potassium ions in a somewhat distorted square planar arrangement [bond angles: 1 *x* 82.67 (1)°, 2 *x* 87.78 (1)°, 1 *x* 101.48 (1)°, bond lengths: 2 *x* 3.1948 (5), 2 *x* 3.1364 (4) Å] with one additional hydrogen atom (H1) to which it forms a hydrogen bond above the potassium plane. The position of this hydrogen atom is not exactly above the chlorine atom but shifted towards the side of the largest K—Cl—K angle.

In the solid (Fig. 5), the arrangement of all components (K^+^, Cl^-^, [SnCl_3_]^-^, H_2_O) is mainly dominated by electrovalent interactions and weak hydrogen bonds to the chlorine atoms as described above. Because various atoms (Sn, 2 *x* Cl, H_2_O) are situated on crystallographic mirror planes, the packing suggest the existence of a layer structure held together through the K^+^ ions between the layers. However, between the building units within the mirror plane there are similar weak interactions as between them and the interlayer atoms.

### Experimental

Colourless, needle-like single crystals of the title compound were prepared on a petri dish within some minutes by adding some drops of an aqueous solution of KCl to solid SnCl_2_.

### Refinement

Our low temperature measurement confirms the formerly determined orthorhombic unit cell but resulted in a smaller unit cell volume at T = 100 K. From systematical absences, space group *Pnma*, the standard setting of space group no 62, was derived. In the original study by Kamenar & Grdenić (1962) the non-standard setting of *Pbnm* was used.

Hydrogen atoms were clearly identified in difference Fourier syntheses. Their positions were refined with respect to a common O—H distance of 0.96 Å and an H—O—H bond angle of 104.9° before they were fixed and allowed to ride on the corresponding oxygen atoms. One common isotropic displacement parameter was refined for both H-atoms.

### Crystal data

**Table:**

K_2_[SnCl_3_]Cl·H_2_O	*F*(000) = 664
*M**_r_* = 356.71	*D*_x_ = 2.678 Mg m^−^^3^
Orthorhombic, *P**n**m**a*	Mo *K*α radiation, λ = 0.71073 Å
Hall symbol: -P 2ac 2n	Cell parameters from 8860 reflections
*a* = 11.9233 (9) Å	θ = 3.0–30.1°
*b* = 9.0768 (7) Å	µ = 4.95 mm^−^^1^
*c* = 8.1737 (6) Å	*T* = 100 K
*V* = 884.60 (12) Å^3^	Needle, colourless
*Z* = 4	0.24 × 0.06 × 0.04 mm

### Data collection

**Table:**

Bruker APEXII CCD diffractometer	1367 independent reflections
Radiation source: fine-focus sealed tube	1265 reflections with *I* > 2σ(*I*)
Graphite monochromator	*R*_int_ = 0.052
φ and ω scans	θ_max_ = 30.0°, θ_min_ = 3.0°
Absorption correction: multi-scan (*SADABS*; Bruker, 2009)	*h* = −16→16
*T*_min_ = 0.388, *T*_max_ = 0.823	*k* = −12→12
27095 measured reflections	*l* = −11→11

### Refinement

**Table:**

Refinement on *F*^2^	Secondary atom site location: difference Fourier map
Least-squares matrix: full	Hydrogen site location: inferred from neighbouring sites
*R*[*F*^2^ > 2σ(*F*^2^)] = 0.013	H-atom parameters constrained
*wR*(*F*^2^) = 0.028	*w* = 1/[σ^2^(*F*_o_^2^) + (0.0078*P*)^2^ + 0.3172*P*] where *P* = (*F*_o_^2^ + 2*F*_c_^2^)/3
*S* = 1.12	(Δ/σ)_max_ = 0.002
1367 reflections	Δρ_max_ = 0.34 e Å^−^^3^
45 parameters	Δρ_min_ = −0.48 e Å^−^^3^
0 restraints	Extinction correction: *SHELXL97* (Sheldrick, 2008), Fc^*^=kFc[1+0.001xFc^2^λ^3^/sin(2θ)]^-1/4^
Primary atom site location: structure-invariant direct methods	Extinction coefficient: 0.00216 (14)

### Special details

**Table:**

Geometry. All e.s.d.'s (except the e.s.d. in the dihedral angle between two l.s. planes) are estimated using the full covariance matrix. The cell e.s.d.'s are taken into account individually in the estimation of e.s.d.'s in distances, angles and torsion angles; correlations between e.s.d.'s in cell parameters are only used when they are defined by crystal symmetry. An approximate (isotropic) treatment of cell e.s.d.'s is used for estimating e.s.d.'s involving l.s. planes.
Refinement. Refinement of *F*^2^ against ALL reflections. The weighted *R*-factor *wR* and goodness of fit *S* are based on *F*^2^, conventional *R*-factors *R* are based on *F*, with *F* set to zero for negative *F*^2^. The threshold expression of *F*^2^ > σ(*F*^2^) is used only for calculating *R*-factors(gt) *etc*. and is not relevant to the choice of reflections for refinement. *R*-factors based on *F*^2^ are statistically about twice as large as those based on *F*, and *R*- factors based on ALL data will be even larger.

### Fractional atomic coordinates and isotropic or equivalent isotropic displacement parameters (Å^2^)

**Table:**

	*x*	*y*	*z*	*U*_iso_*/*U*_eq_	
Sn1	1.010043 (11)	0.7500	−0.009701 (14)	0.00860 (5)	
Cl1	0.80674 (4)	0.7500	0.10649 (5)	0.01085 (9)	
Cl2	1.05741 (3)	0.55307 (3)	0.20619 (4)	0.01175 (7)	
Cl3	0.73496 (4)	0.2500	0.10123 (5)	0.01180 (9)	
K1	0.81583 (3)	0.48246 (3)	0.36996 (4)	0.01235 (7)	
O1	0.96072 (13)	0.2500	0.44548 (17)	0.0146 (3)	
H1	1.0397	0.2500	0.4228	0.070 (8)*	
H2	0.9554	0.2500	0.5627	0.070 (8)*	

### Atomic displacement parameters (Å^2^)

**Table:**

	*U*^11^	*U*^22^	*U*^33^	*U*^12^	*U*^13^	*U*^23^
Sn1	0.00816 (8)	0.00788 (7)	0.00976 (7)	0.000	0.00094 (4)	0.000
Cl1	0.0081 (2)	0.01165 (19)	0.0128 (2)	0.000	0.00042 (15)	0.000
Cl2	0.01092 (17)	0.01012 (14)	0.01423 (15)	0.00051 (11)	−0.00054 (11)	0.00207 (11)
Cl3	0.0123 (2)	0.01050 (19)	0.0126 (2)	0.000	−0.00049 (16)	0.000
K1	0.01198 (16)	0.01039 (13)	0.01469 (14)	0.00027 (10)	0.00196 (10)	−0.00046 (10)
O1	0.0131 (8)	0.0174 (7)	0.0132 (7)	0.000	0.0007 (5)	0.000

### Geometric parameters (Å, º)

**Table:**

Sn1—Cl2	2.5745 (3)	Cl3—K1^ii^	3.1364 (4)
Sn1—Cl2^i^	2.5745 (3)	Cl3—K1	3.1948 (5)
Sn1—Cl1	2.6034 (5)	Cl3—K1^vi^	3.1948 (5)
Cl1—K1^ii^	3.2134 (5)	K1—O1	2.7960 (10)
Cl1—K1^iii^	3.2134 (5)	K1—Cl3^vii^	3.1364 (4)
Cl1—K1	3.2475 (4)	K1—Cl2^viii^	3.2081 (5)
Cl1—K1^i^	3.2476 (4)	K1—Cl1^ix^	3.2133 (5)
Cl2—K1^iv^	3.2081 (5)	O1—K1^vi^	2.7960 (10)
Cl2—K1	3.2403 (5)	O1—H1	0.9600
Cl3—K1^v^	3.1364 (4)	O1—H2	0.9600
			
Cl2—Sn1—Cl2^i^	87.945 (16)	O1—K1—Cl2^viii^	141.91 (3)
Cl2—Sn1—Cl1	87.376 (11)	Cl3^vii^—K1—Cl2^viii^	77.105 (12)
Cl2^i^—Sn1—Cl1	87.376 (11)	Cl3—K1—Cl2^viii^	73.061 (12)
Sn1—Cl1—K1^ii^	101.770 (13)	O1—K1—Cl1^ix^	69.67 (3)
Sn1—Cl1—K1^iii^	101.770 (13)	Cl3^vii^—K1—Cl1^ix^	93.324 (13)
K1^ii^—Cl1—K1^iii^	82.088 (15)	Cl3—K1—Cl1^ix^	80.950 (12)
Sn1—Cl1—K1	102.172 (12)	Cl2^viii^—K1—Cl1^ix^	79.112 (12)
K1^ii^—Cl1—K1	85.592 (7)	O1—K1—Cl2	72.01 (3)
K1^iii^—Cl1—K1	154.844 (16)	Cl3^vii^—K1—Cl2	105.515 (13)
Sn1—Cl1—K1^i^	102.171 (12)	Cl3—K1—Cl2	96.598 (13)
K1^ii^—Cl1—K1^i^	154.844 (16)	Cl2^viii^—K1—Cl2	137.232 (12)
K1^iii^—Cl1—K1^i^	85.592 (7)	Cl1^ix^—K1—Cl2	141.509 (13)
K1—Cl1—K1^i^	96.794 (16)	O1—K1—Cl1	137.00 (3)
Sn1—Cl2—K1^iv^	102.498 (12)	Cl3^vii^—K1—Cl1	79.297 (12)
Sn1—Cl2—K1	103.031 (13)	Cl3—K1—Cl1	91.597 (13)
K1^iv^—Cl2—K1	153.434 (14)	Cl2^viii^—K1—Cl1	71.936 (12)
K1^v^—Cl3—K1^ii^	101.475 (17)	Cl1^ix^—K1—Cl1	151.022 (11)
K1^v^—Cl3—K1	169.741 (15)	Cl2—K1—Cl1	66.909 (11)
K1^ii^—Cl3—K1	87.782 (6)	K1^vi^—O1—K1	97.99 (5)
K1^v^—Cl3—K1^vi^	87.782 (7)	K1^vi^—O1—H1	124.3
K1^ii^—Cl3—K1^vi^	169.741 (15)	K1—O1—H1	124.3
K1—Cl3—K1^vi^	82.669 (16)	K1^vi^—O1—H2	100.3
O1—K1—Cl3^vii^	124.79 (3)	K1—O1—H2	100.3
O1—K1—Cl3	80.79 (3)	H1—O1—H2	104.9
Cl3^vii^—K1—Cl3	150.166 (11)		

Symmetry codes: (i) *x*, −*y*+3/2, *z*; (ii) −*x*+3/2, −*y*+1, *z*−1/2; (iii) −*x*+3/2, *y*+1/2, *z*−1/2; (iv) *x*+1/2, *y*, −*z*+1/2; (v) −*x*+3/2, *y*−1/2, *z*−1/2; (vi) *x*, −*y*+1/2, *z*; (vii) −*x*+3/2, *y*+1/2, *z*+1/2; (viii) *x*−1/2, *y*, −*z*+1/2; (ix) −*x*+3/2, *y*−1/2, *z*+1/2.

### Hydrogen-bond geometry (Å, º)

**Table:**

*D*—H···*A*	*D*—H	H···*A*	*D*···*A*	*D*—H···*A*
O1—H1···Cl3^x^	0.96	2.34	3.2920 (16)	174
O1—H2···Cl2^xi^	0.96	2.61	3.3687 (12)	137
O1—H2···Cl2^xii^	0.96	2.61	3.3687 (12)	137

Symmetry codes: (x) *x*+1/2, −*y*+1/2, −*z*+1/2; (xi) −*x*+2, −*y*+1, −*z*+1; (xii) −*x*+2, *y*−1/2, −*z*+1.
